# The Compounded Risk of Maternal Anemia and Preeclampsia: Neonatal Outcomes and Predictive Modeling in a Low-Resource Tertiary Center

**DOI:** 10.3390/jcm14145051

**Published:** 2025-07-16

**Authors:** Victor Bogdan Buciu, Sebastian Ciurescu, Denis Mihai Șerban, Dorin Novacescu, Nicolae Nicoleta, Larisa Tomescu, Elena Lavinia Rusu, Ioan Sas, Mihai Ionac, Veronica-Daniela Chiriac

**Affiliations:** 1 Doctoral School, “Victor Babes” University of Medicine and Pharmacy, E. Murgu Square, No. 2, 300041 Timisoara, Romania; victor.buciu@umft.ro (V.B.B.);; 2 Department of Obstetrics-Gynaecology, Discipline of Obstetrics-Gynecology, “Victor Babes” University of Medicine and Pharmacy, E. Murgu Square, No. 2, 300041 Timisoara, Romania; denis.serban@umft.ro (D.M.Ș.); nicolae.nicoleta@umft.ro (N.N.); tomescu.larisa@umft.ro (L.T.); sas.ioan@umft.ro (I.S.); chiriac.veronica@umft.ro (V.-D.C.); 3 Department of Microscopic Morphology, Discipline of Histology, “Victor Babes” University of Medicine and Pharmacy, E. Murgu Square, No. 2, 300041 Timisoara, Romania; novacescu.dorin@umft.ro; 4 Department of Microsurgery, Vascular Surgery and Scientific Research Methodology, “Victor Babes” University of Medicine and Pharmacy, E. Murgu Square, No. 2, 300041 Timisoara, Romania; mihai.ionac@gmail.com

**Keywords:** preeclampsia, maternal anemia, neonatal outcomes, preterm birth, NICU admission, hemoglobin levels, perinatal morbidity

## Abstract

**Background**: Anemia and preeclampsia are common and independently associated with adverse neonatal outcomes. Their combined effect, however, remains insufficiently explored. This study aims to evaluate the impact of second-trimester maternal anemia on neonatal outcomes in pregnancies complicated by preeclampsia, and to assess its predictive value for preterm birth and NICU admission. **Methods**: We conducted a retrospective cohort study including 3517 singleton births from a Romanian tertiary maternity hospital between October 2023 and December 2024. A total of 295 preeclamptic pregnancies were stratified by anemia severity (none, mild, moderate-to-severe) and compared with 428 matched non-preeclamptic anemic pregnancies matched by closest-neighbor selection. Neonatal outcomes included birthweight, gestational age, anthropometric parameters, Apgar score, preterm birth, and NICU admission. Logistic regression and ROC curve analysis were performed using anemia severity as a predictor. **Results**: Moderate-to-severe anemia in preeclamptic pregnancies was associated with significantly lower birthweight (2618 ± 461 g), shorter gestational age (36.6 ± 2.0 weeks), and higher preterm birth (41.1%) and NICU admission rates (40.0%) were compared to non-anemic counterparts. Each increase in anemia severity conferred 84% higher odds of preterm delivery (OR = 1.84; AUC = 0.63) and a 49% increase in NICU admission (OR = 1.49; AUC = 0.58). Youden’s indices were 0.25 and 0.14, respectively. **Conclusions**: Maternal anemia is associated with increased neonatal morbidity in preeclamptic pregnancies, with moderate predictive value for preterm birth. These findings support the integration of early anemia screening and risk stratification into hypertensive pregnancy protocols to improve perinatal outcomes.

## 1. Introduction

Preeclampsia remains one of the leading causes of maternal and perinatal morbidity and mortality worldwide, complicating approximately 5–8% of pregnancies and contributing substantially to preterm birth, intrauterine growth restriction, and stillbirth. In Romania, the reported prevalence of preeclampsia varies between 2.5% and 6.2%, depending on the population studied and diagnostic criteria applied, while maternal anemia affects approximately 28–35% of pregnancies, with higher rates in socioeconomically disadvantaged groups [1]. Characterized by hypertension and end-organ dysfunction after 20 weeks of gestation, preeclampsia is rooted in defective placentation, systemic endothelial injury, and imbalances in angiogenic signaling [1,2,3].

Anemia in pregnancy—defined by the World Health Organization as hemoglobin levels below 11 g/dL—also affects a large proportion of expectant mothers globally, with prevalence estimates ranging from 20% to over 50% depending on region and socioeconomic context. Iron-deficiency anemia, the most common subtype, is associated with increased risk for low birth weight, preterm delivery, and neonatal intensive care unit (NICU) admission [4,5,6,7].

While both preeclampsia and anemia independently impact fetal well-being, their interaction is insufficiently understood. A limited number of studies have explored how anemia modifies the course and severity of preeclampsia, and even fewer have focused on how this interplay affects neonatal outcomes. Given that both conditions compromise placental oxygen and nutrient delivery—albeit through distinct mechanisms—their coexistence may exert a compounded effect on fetal growth and neonatal adaptation [8,9,10].

Importantly, anemia is typically detected during routine second-trimester screening (24–28 weeks), coinciding with glucose tolerance testing and other maternal assessments. This window offers an opportunity for early identification of hematologic risk factors in pregnancies that may later develop hypertensive complications.

This study aims to quantify the extent to which maternal anemia at 24–28 weeks exacerbates the adverse neonatal outcomes already associated with preeclampsia, and secondarily, to determine whether these outcomes differ from those of anemic pregnancies without preeclampsia. We hypothesized that anemia would exacerbate the adverse neonatal outcomes already associated with preeclampsia.

## 2. Materials and Methods

### 2.1. Study Design

This retrospective observational study was conducted at a single tertiary care maternity hospital in Romania and covered all singleton births recorded between 1 October 2023 and 31 December 2024. The study was designed to evaluate the impact of maternal anemia—classified by severity—on neonatal outcomes in pregnancies affected by preeclampsia, and secondarily to determine whether these outcomes differed from those of pregnancies with anemia but without preeclampsia. Institutional ethical approval was obtained prior to study initiation (Approval No. 78, date 2 October 2023).

All patient records were screened retrospectively from institutional databases, identifying 3517 deliveries within the study period. All values were extracted and verified by two independent investigators. All data were fully anonymized in accordance with institutional policy and the General Data Protection Regulation (EU) 2016/679 [11]. Patient identifiers were removed, and only authorized researchers accessed the final dataset.

### 2.2. Patient Selection and Grouping

Patients were selected if they met the diagnostic criteria for preeclampsia according to the 2018 American College of Obstetricians and Gynecologists (ACOG) guidelines [12]. For each case, relevant clinical data were extracted, including maternal age, body mass index (BMI), parity, diabetes status, and hemoglobin concentration between 24 and 28 weeks of gestation. Neonatal data were collected simultaneously, including gestational age at birth, birthweight, birth length, APGAR scores, and NICU admission status.

Anemia severity was defined based on World Health Organization cutoffs. Gestational age was determined by early first-trimester crown–rump length or last menstrual period and later confirmed postpartum during specialist pediatric examination.

To minimize confounding due to comorbid conditions, exclusions were applied to patients with severe underlying maternal disease involving any organ system. This included documented cases of advanced chronic kidney disease, autoimmune conditions such as systemic lupus erythematosus, chronic liver disease, congestive heart failure, and any form of active malignancy. Multiple gestation, known fetal anomalies, and intrauterine fetal demise were also excluded.

Additionally, patients who received iron supplementation or anemia correction before 32 weeks were excluded to avoid treatment-related confounding. This exclusion was justified by literature indicating that iron correction late in pregnancy has a limited effect on neonatal outcomes, but remains more significant the earlier it is introduced [13,14,15,16].

After data acquisition, we have followed a two-stage design to evaluate how second-trimester anemia affects neonatal outcomes, both in pregnancies with and without preeclampsia. In the first stage, women with preeclampsia were stratified into three groups: no anemia, mild anemia (Hb 10.0–10.9 g/dL), and moderate-to-severe anemia (Hb < 10.0 g/dL). In the second stage, a control group—selected based on a closest-neighbor strategy—without preeclampsia but with anemia was classified into mild anemia and moderate-to-severe anemia, using the same thresholds.

This structure enabled two key comparisons: within the preeclampsia cohort, to assess how anemia severity influences neonatal outcomes; and between matched anemia groups with and without preeclampsia, to evaluate the additive effect of preeclampsia on fetal risk.

To minimize bias and enhance comparability between groups, we applied a closest-neighbor selection strategy based on key maternal parameters, including age, BMI, parity, diabetes status, and conception method.

A 1:1.5 matching ratio was chosen based on the availability of eligible non-preeclamptic anemic pregnancies in our institutional database, which exceeded the number of preeclamptic cases. This allowed us to increase the control group size while maintaining rigorous matching quality. Given that we applied strict caliper constraints and excluded poorly matched controls, the 1:1.5 ratio represented an optimal balance between statistical power and comparability. In our center, this approach is particularly valuable due to the relatively high prevalence of anemia and the moderate volume of hypertensive pregnancies. The slightly larger control group improves the precision of estimates while preserving internal validity through tight matching on clinically relevant covariates.

### 2.3. Statistical Analysis

Descriptive statistics were used to characterize the maternal and neonatal profiles across the five groups. Continuous variables were expressed as means and standard deviations. Group comparisons for continuous variables were performed using one-way analysis of variance (ANOVA), followed by Tukey’s honestly significant difference (HSD) test for post hoc pairwise comparisons. For categorical variables, Chi-square or Fisher’s exact tests were used where appropriate. A *p*-value of <0.05 was considered statistically significant for overall ANOVA, and individual post hoc comparisons were interpreted based on Tukey-adjusted *p*-values.

Apgar score at 5 min was analyzed as a categorical variable. We calculated the proportion of neonates with scores <7 and <5, and compared these proportions across study groups using the Chi-square test. Median and interquartile ranges were also considered, but the percentage of low scores was deemed more clinically relevant.

A two-tailed *p*-value of less than 0.05 was considered statistically significant for all tests. Statistical analyses were performed using IBM SPSS Statistics version 28.0. All statistical procedures adhered to the STROBE (Strengthening the Reporting of Observational Studies in Epidemiology) recommendations for observational cohort studies.

## 3. Results

### 3.1. Grouping of the Study Population

Between 1 October 2023 and 31 December 2024, a total of 3517 singleton deliveries were recorded at our tertiary maternity hospital. All cases were retrospectively screened for eligibility. Among these, 614 pregnancies met the diagnostic criteria for preeclampsia according to the 2018 ACOG guidelines [12]. A series of exclusion criteria was subsequently applied to ensure analytical consistency and data integrity. Specifically, 191 pregnancies were excluded due to missing hemoglobin values between 24 and 28 weeks of gestation, 48 cases were excluded because anemia correction (either oral or parenteral) had been initiated before 32 weeks, and 38 cases involved severe maternal comorbidities such as advanced renal disease, autoimmune disorders, chronic liver disease, or malignancy. An additional 19 pregnancies were excluded due to major fetal anomalies or intrauterine demise, while 23 cases were removed due to incomplete neonatal outcome data. Following these exclusions, 295 preeclamptic pregnancies were retained for final analysis.

To enable a rigorous group comparison analysis, we selected control groups based on a nearest-neighbor selection process, as previously described in Section 2.2, on a predetermined ratio. As a result, a final cohort of 428 non-preeclamptic pregnancies with anemia was identified that was clinically and demographically comparable to the 295 preeclamptic cases.

We opted for tight caliper matching to enhance methodological precision and excluded unmatched or poorly matched control cases, even at the expense of a reduced sample size. Matching was conducted using a nearest-neighbor algorithm without replacement, applying a caliper width of 0.2 standard deviations of the propensity score. Post-matching balance was confirmed, with all covariates achieving a standardized mean difference (SMD) below 0.1, indicating strong comparability between the groups.

**Figure 1:**
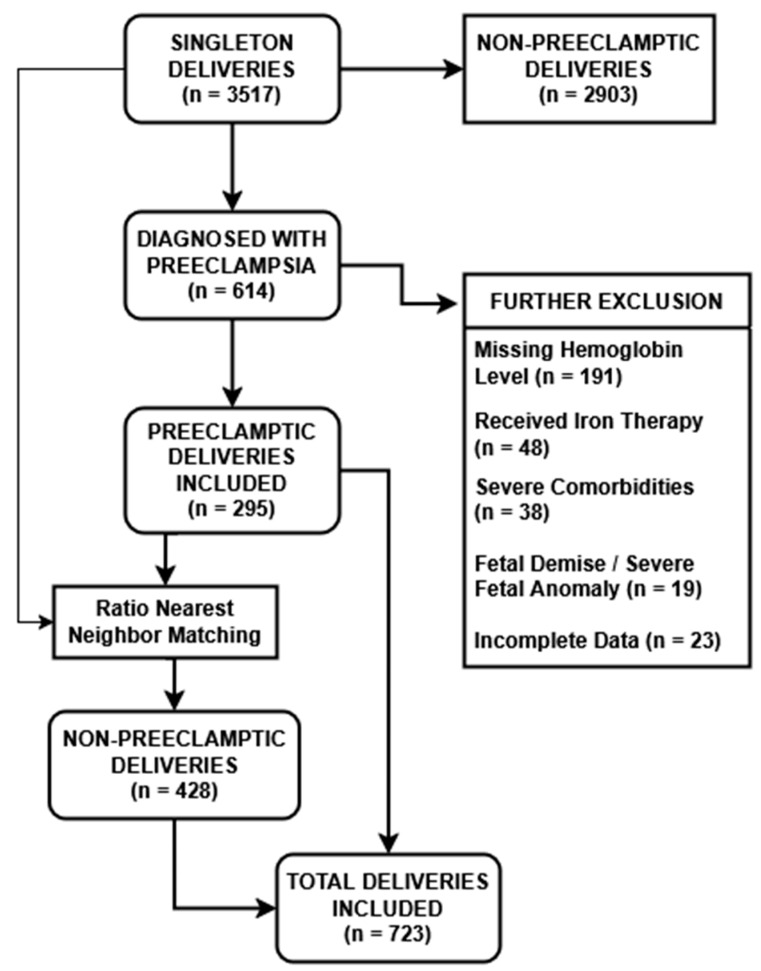
PRISMA-like Patient Selection Process.

The complete patient selection process is illustrated in Figure 1.

A total of 723 pregnancies met the inclusion criteria and were categorized into five study groups based on preeclampsia status and hemoglobin values, as presented in Table 1. Among the 295 pregnancies complicated by preeclampsia, 91 women had no anemia (PE, no anemia), 109 had mild anemia (PE, mild anemia), and 95 had moderate-to-severe anemia (PE, mod–severe anemia). In the non-preeclamptic population, 234 women had mild anemia (No PE, mild anemia), and 194 had moderate-to-severe anemia (No PE, mod–severe anemia).

### 3.2. Maternal Results

To contextualize baseline variability, maternal demographic parameters revealed modest but statistically relevant differences across the five study groups, as outlined in Table 2.

Maternal variables analyzed included age, BMI, parity, smoking status, diabetes mellitus, and chronic hypertension. Smoking history was available for all patients and is summarized in Table 2. These variables were also used in the matching algorithm to ensure baseline comparability.

Women with preeclampsia were generally older and exhibited higher BMI compared to their non-preeclamptic counterparts. The average maternal age in preeclamptic groups ranged from 30.6 to 31.0 years, while in non-preeclamptic groups it remained slightly lower, around 29.8 years. Similarly, BMI was consistently higher in preeclamptic pregnancies (mean approx. 27.2–27.4 kg/m^2^) compared to non-preeclamptic pregnancies (approx. 25.9–26.0 kg/m^2^), aligning with previous evidence that elevated BMI is a known risk factor for hypertensive disorders in pregnancy.

Parity was marginally lower in the preeclamptic population (mean parity approx. 1.0–1.1), suggesting a higher proportion of nulliparous women, which is consistent with epidemiological trends in preeclampsia.

Smoking was slightly more common in the PE No Anemia group (18%) compared to other groups, though it remained within the expected population levels. Importantly, the prevalence of chronic comorbidities such as diabetes and chronic hypertension was elevated in preeclamptic pregnancies, particularly in those with anemia. For example, chronic hypertension was present in 12–14% of anemic preeclamptic women compared to just 4% in non-preeclamptic anemic groups, reinforcing the notion that systemic vascular dysfunction may underlie or exacerbate the development of preeclampsia.

These maternal factors—particularly smoking and preexisting hypertension—are well-documented contributors to adverse perinatal outcomes and were therefore included in both descriptive and comparative analyses [17,18].

Taken together, these demographic patterns suggest that women with preeclampsia, especially those with concurrent anemia, represent a more medically complex subgroup with higher baseline cardiovascular and metabolic risk profiles. These background characteristics may partially explain the compounded adverse fetal outcomes observed in this population.

### 3.3. Neonatal Outcomes by Group

Neonatal outcomes varied significantly across the five study groups, reflecting the combined and individual influences of maternal anemia and preeclampsia. Table 3 summarizes the birthweight, gestational age, anthropometric measurements, and early neonatal adaptation parameters according to anemia severity and preeclampsia status.

Neonatal outcomes demonstrated a clear gradient of worsening with increasing severity of maternal anemia, most notably when preeclampsia was also present. Among all study groups, infants born to mothers with preeclampsia and moderate-to-severe anemia exhibited the most compromised clinical profile, including significantly lower birthweight, reduced anthropometric measurements, earlier gestational age at delivery, and the highest rates of preterm birth and NICU admission. The proportion of neonates with 5 min Apgar scores <7 was highest in the PE with moderate-to-severe anemia group (12.6%), compared to 3.2% in the PE without anemia group and 2.6% in non-preeclamptic mildly anemic pregnancies (*p* < 0.01, Chi-square test). This supports a trend of worsening neonatal adaptation in the presence of both anemia and preeclampsia. These patterns were not only statistically significant but clinically meaningful, reflecting a heightened vulnerability of the fetus in the dual presence of maternal hypertension and anemia.

Even in the context of mild anemia, preeclamptic pregnancies showed measurable impairment in neonatal parameters compared to non-anemic counterparts, highlighting that even subtle reductions in hemoglobin may aggravate placental dysfunction. In the absence of preeclampsia, moderate-to-severe anemia was also associated with adverse outcomes, although the magnitude of effect was consistently less than that observed in anemic preeclamptic pregnancies. This contrast reinforces the concept of a pathophysiological synergy between anemia and preeclampsia, in which their coexistence intensifies placental compromise beyond the contribution of either factor alone.

Conversely, the most favorable neonatal outcomes were observed in women without preeclampsia and only mild anemia, as well as in non-anemic preeclamptic patients, suggesting that maintaining hemoglobin levels above critical thresholds may buffer against fetal compromise, even in high-risk pregnancies. These findings underscore the importance of early anemia screening and severity stratification, particularly in women already predisposed to hypertensive complications.

### 3.4. Pairwise Comparison Analysis

To evaluate the specific between-group differences in neonatal outcomes, we performed one-way ANOVA followed by Tukey’s honestly significant difference (HSD) post hoc test for all continuous variables. The overall ANOVA results indicated statistically significant differences across the five groups for birthweight, gestational age, birth length, and head circumference (all *p* < 0.001). Post hoc pairwise comparisons further clarified the source of these differences.

Neonates born to mothers with preeclampsia and moderate-to-severe anemia had significantly lower birthweight and shorter gestational age compared to all other groups (*p* < 0.001 for comparisons with both non-anemic and mildly anemic preeclamptic patients, as well as with non-preeclamptic anemic controls). This group also exhibited the most compromised anthropometric parameters, including significantly reduced length and head circumference.

Among patients with mild anemia, those with coexisting preeclampsia had significantly lower birthweight and gestational age compared to non-preeclamptic anemic controls (*p* < 0.01), reinforcing the additional burden conferred by hypertensive disease. Similarly, when comparing moderate-to-severe anemia with and without preeclampsia, neonates from the preeclamptic group had significantly worse outcomes across all major parameters.

These results highlight the compounding effect of preeclampsia and anemia, with the most severe neonatal compromise observed when both conditions were present. The findings remained robust after correction for multiple comparisons, supporting the validity of the observed trends.

### 3.5. Anemia Severity as Predictive Factor for Neonatal Outcomes

To assess the predictive utility of maternal anemia severity in preeclamptic pregnancies, two logistic regression models were constructed—one for preterm birth and the other for NICU admission—as binary outcomes. In both models, anemia severity was the sole independent variable, entered as an ordinal predictor coded from 0 (no anemia) to 2 (moderate-to-severe anemia).

In the model predicting preterm birth, increasing anemia severity was associated with significantly elevated odds of delivery before 37 weeks (OR = 1.84; 95% CI: 1.27–2.66; *p* = 0.001). The model demonstrated moderate discriminative power, with an area under the receiver operating characteristic curve (AUC) of 0.63. The optimal threshold probability for predicting preterm delivery was 0.400, yielding a sensitivity of 50.0%, a specificity of 74.6%, and a Youden index of 0.25.

In the NICU admission model, anemia severity also showed a significant association (OR = 1.49; 95% CI: 1.04–2.13; *p* = 0.030), though the model’s discriminative ability was more modest (AUC = 0.58). The optimal probability cutoff for NICU admission prediction was 0.369, corresponding to a sensitivity of 42.4%, specificity of 71.9%, and Youden index of 0.14.

Overall, these models confirm that maternal anemia contributes to neonatal risk among preeclamptic pregnancies, particularly with regard to early delivery. While its predictive power for NICU admission is more modest, the direction and consistency of the associations underscore anemia’s potential value as a prognostic indicator.

## 4. Discussion

### 4.1. Principal Findings

This study provides compelling evidence that maternal anemia, particularly of moderate-to-severe intensity, is significantly associated with adverse neonatal outcomes in pregnancies complicated by preeclampsia. The combination of these two conditions was associated with substantial reductions in birthweight and gestational age, as well as increased rates of preterm birth and NICU admissions.

To our knowledge, this is the first study to quantify the predictive value of maternal anemia severity on neonatal outcomes specifically within a preeclamptic population, using both odds ratios and ROC curve analysis. While prior research has established independent associations between anemia and adverse perinatal outcomes, our study uniquely isolates anemia’s additive risk in the context of hypertensive pregnancy. By demonstrating a stepwise increase in the odds of preterm birth and NICU admission with worsening anemia severity—and by reporting corresponding AUC and Youden indices—this analysis offers a novel risk stratification framework that could inform both clinical surveillance and early intervention strategies in high-risk obstetric care.

Our findings align with previous research indicating that maternal anemia is a significant risk factor for adverse perinatal outcomes. A systematic review and meta-analysis found that maternal anemia increased the risk of low birth weight (OR 1.65), preterm birth (OR 2.11), and perinatal mortality (OR 3.01). Similarly, they reported that both low and high maternal hemoglobin concentrations during pregnancy are strong predictors of adverse maternal and infant health outcomes [19,20]. Numerous studies have independently associated maternal anemia with increased risks of low birth weight, preterm birth, and perinatal mortality [21].

The interplay between anemia and preeclampsia has been less extensively studied. Our study contributes to this gap by demonstrating that the coexistence of these two conditions exacerbates adverse neonatal outcomes more than either condition alone. Our findings are supported by research indicating that the combination of anemia and hypertensive disorders during pregnancy leads to worse outcomes. For instance, a study observed that anemia in the third trimester is associated with increased risks of low birth weight and preterm delivery [22]. This synergistic effect is supported by a study conducted in Southern Ethiopia, which found that maternal anemia was associated with increased risks of low birth weight (aRR = 3.02) and respiratory distress syndrome (aRR = 4.82) in neonates [23]. Additionally, a study reported that iron-deficiency anemia during pregnancy is linked to higher incidences of preterm birth and NICU admissions [24]. Our findings are also consistent with another paper, which found that the baby birth weight decreased significantly with the decrease in maternal hemoglobin levels [25]. Lastly, the impact of maternal anemia on neonatal hemoglobin levels has been highlighted in a meta-analysis, which confirmed that maternal anemia during pregnancy increases the risk of low neonatal hemoglobin levels [26].

In association with its treatment, oral iron therapy within anemic pregnancies has been associated with a reduction in the odds of preterm birth and preeclampsia. A study found that successfully treated patients with anemia had a significant reduction in the odds of preterm birth (aOR 0.59) and preeclampsia (aOR 0.75) [14]. This emphasizes the importance of early detection and management of anemia during pregnancy.

### 4.2. Potential Mechanisms

The compounded adverse neonatal outcomes observed in pregnancies complicated by both anemia and preeclampsia can be attributed to overlapping and synergistic pathophysiological mechanisms that impair oxygen delivery and placental function.

Maternal anemia, characterized by reduced hemoglobin levels, diminishes the oxygen-carrying capacity of the blood, leading to decreased oxygen delivery to the placenta and fetus [24]. This hypoxic environment can impair fetal growth and development and increase the risk of newborn anemia, as confirmed in prior studies [9,24,25,26]. Preeclampsia is associated with abnormal placental development, particularly inadequate remodeling of the spiral arteries, leading to high-resistance blood flow and placental ischemia. This results in reduced nutrient and oxygen delivery to the fetus, contributing to fetal growth restriction [27,28,29], secondary to impaired vascularization [30,31]. Both anemia and preeclampsia are associated with increased oxidative stress. In anemia, hypoxia can lead to the generation of reactive oxygen species, while in preeclampsia, placental ischemia and reperfusion injury exacerbate ROS production and mitochondrial dysfunction [23,24,25,26]. The combination of these conditions may amplify oxidative damage, further compromising placental function and fetal development.

In addition, preeclampsia involves systemic endothelial dysfunction and an imbalance of angiogenic factors, such as increased levels of sFlt-1, which antagonizes VEGF and PlGF, further impairing placental vascularization [29,30,31]. This proinflammatory environment contributes to widespread endothelial activation and vascular injury. Anemia may further exacerbate these inflammatory cascades through both iron deficiency-related immunologic effects and secondary responses to tissue hypoxia [29,30]. When maternal anemia and preeclampsia coexist, their effects on oxygen delivery and placental function are compounded. The reduced oxygen-carrying capacity due to anemia, combined with the impaired placental perfusion from preeclampsia, creates a severely hypoxic intrauterine environment [27,28,29,30,31]. This exacerbates fetal growth restriction and increases the risk and increases the risk of preterm birth and other adverse neonatal outcomes [10,27,28].

### 4.3. Clinical Implications

Routine screening for anemia during pregnancy is a standard practice; however, our results suggest that more vigilant monitoring is warranted, especially in patients with or at risk for preeclampsia. Healthcare providers should develop integrated care plans that address both conditions simultaneously, considering the potential for compounded effects on fetal development. A prior risk-based stratification for preeclampsia development was suggested and utilized in guiding clinical treatment and follow-up [29].

Addressing nutritional deficiencies through dietary counseling and supplementation is crucial. In cases of iron-deficiency anemia, appropriate iron supplementation should be initiated promptly [15,30]. For patients with preeclampsia, antihypertensive therapy and close monitoring are essential. Tailoring interventions to the individual patient’s needs can optimize outcomes.

Educating patients about the risks associated with anemia and preeclampsia, as well as the importance of adherence to treatment regimens, is vital. Empowering patients with knowledge can lead to better compliance and engagement in their care, potentially reducing adverse outcomes. Prior studies highlight that maternal education can have an impact on the risk of severe adverse effects of preeclampsia, and secondarily on the fetal well-being [31]. Interventions aimed at correcting maternal anemia and monitoring for signs of preeclampsia, such as stratifications based on clinical risk or level of health literacy, could mitigate the compounded risks to the fetus and offer time to mitigate potential severe effects.

### 4.4. Limitations

Several methodological limitations should be acknowledged. First, the retrospective design introduces potential biases related to data completeness and documentation quality. Although propensity score matching was used to balance key maternal characteristics (e.g., age, BMI, parity, diabetes), other important confounders such as nutritional status, socioeconomic factors, smoking, and access to prenatal care were not uniformly available and could not be controlled for.

Second, anemia was not stratified by etiology. While iron deficiency is presumed to be the predominant cause, the lack of confirmatory tests (e.g., ferritin, transferrin saturation) limits our ability to differentiate it from other forms such as B12 or folate deficiency, anemia of chronic disease, or hemoglobinopathies. Although we excluded patients with overt systemic disease, undiagnosed or subclinical conditions may have gone undetected.

Third, NICU admission—as an outcome—may be influenced by local institutional policies, staffing, and resource availability, not solely by neonatal severity. At our institution, NICU admission is based on a standardized neonatal triage protocol that includes gestational age below 36 weeks, birthweight below 2500 g, Apgar score ≤7 at 5 min, or presence of respiratory distress, hypoglycemia, or congenital anomalies. However, provider discretion may still influence borderline cases. It should thus be interpreted as a composite indicator reflecting both clinical need and hospital-level decision-making. Moreover, the absence of post-discharge follow-up data prevents assessment of long-term neonatal outcomes related to anemia or preeclampsia.

Finally, this study’s single-center design, while providing protocol consistency, may limit generalizability to other populations with differing healthcare systems, nutritional environments, or genetic backgrounds.

### 4.5. Future Research Directions

The findings of this study raise important questions regarding the intersection of maternal anemia and hypertensive disorders in pregnancy, and they open several avenues for future investigation.

First, prospective multicenter studies with diverse populations are needed to validate the compounded risk posed by anemia and preeclampsia on neonatal outcomes. Expanding beyond a single-center cohort will increase generalizability, account for regional variability in anemia prevalence, and address ethnic, nutritional, and healthcare system differences that may moderate outcomes.

Second, future studies should stratify anemia by etiology, not solely by hemoglobin concentration. Differentiating iron-deficiency anemia from other types (e.g., thalassemia trait, vitamin B12 or folate deficiency, anemia of inflammation) through laboratory biomarkers such as ferritin, transferrin saturation, or CRP could help identify which subtypes are most harmful in the context of preeclampsia and tailor interventions accordingly.

Third, more attention should be paid to temporal trends in hemoglobin levels. While our study captured anemia at 24–28 weeks, longitudinal data capturing first, second, and third trimester hemoglobin trajectories could clarify critical windows for intervention.

Fourth, the role of anemia correction therapies deserves greater scrutiny. Randomized controlled trials examining the impact of early vs. late iron supplementation, parenteral iron use, or dietary interventions in women at risk for preeclampsia could provide direct evidence for clinical guidelines.

Finally, from a public health perspective, research should explore cost-effective screening and intervention strategies for anemia and preeclampsia in low- and middle-income countries, where both conditions are prevalent and often underdiagnosed.

## 5. Conclusions

This retrospective cohort study demonstrates that maternal anemia, particularly of moderate-to-severe intensity, is significantly associated with worsened neonatal outcomes in pregnancies complicated by preeclampsia. These effects include reduced birthweight, shortened gestational age, and increased risks of preterm delivery and NICU admission. When anemia coexists with preeclampsia, the compounded burden on fetal well-being becomes especially apparent, highlighting a critical area for clinical vigilance.

Importantly, our findings reveal that even in the absence of preeclampsia, moderate-to-severe maternal anemia is independently associated with adverse neonatal metrics—though to a lesser extent. This comparative insight reinforces the idea that anemia is not merely a co-variable but a significant perinatal risk factor in its own right.

By integrating a structured group design with clinically relevant variables, and by controlling for treatment exposure, this study contributes meaningful evidence to the growing recognition of anemia’s role in high-risk obstetrics. These results advocate for more proactive screening, etiological classification, and management of anemia during pregnancy, especially in patients with hypertensive disorders.

Further research—both prospective and mechanistic—is needed to refine risk prediction and intervention strategies.

## Figures and Tables

**Table 1:** Study Group Definitions and Sample Sizes.

Group	Condition	Hemoglobin at 24–28 Weeks	Number of Patients
PE, no anemia	Preeclampsia	≥11 g/dL	91
PE, mild anemia	Preeclampsia	10.0–10.9 g/dL	109
PE, mod–severe anemia	Preeclampsia	<10.0 g/dL	95
No PE, mild anemia	No preeclampsia	10.0–10.9 g/dL	234
No PE, mod–severe anemia	No preeclampsia	<10.0 g/dL	194

Hemoglobin (Hb) levels were measured between 24 and 28 weeks of gestation. Anemia was classified according to World Health Organization (WHO) criteria: mild anemia as Hb 10.0–10.9 g/dL and moderate-to-severe anemia as Hb < 10.0 g/dL. Patients receiving anemia treatment before 32 weeks were excluded to avoid treatment-related confounding. PE = preeclampsia.

**Table 2:** Maternal Characteristics by Study Group.

Maternal Characteristic	No PE, Mild Anemia	No PE, Mod–Severe Anemia	PE, No Anemia	PE, Mild Anemia	PE, Mod–Severe Anemia
N	234	194	91	109	95
Maternal Age (years)	29.90 ± 3.83	29.75 ± 3.90	30.61 ± 3.72	30.96 ± 4.27	30.76 ± 3.94
BMI (kg/m^2^)	25.95 ± 2.86	25.90 ± 3.01	27.15 ± 2.90	27.19 ± 2.82	27.39 ± 3.06
Parity	1.23 ± 1.18	1.21 ± 0.98	1.04 ± 1.09	1.06 ± 1.13	1.01 ± 0.89
Smoking (%)	12%	12%	18%	10%	9%
Diabetes Mellitus (%)	7%	9%	9%	11%	13%
Chronic Hypertension (%)	4%	4%	8%	14%	12%

Values are presented as mean ± standard deviation or percentage of group total. PE = preeclampsia; HTN = hypertension; BMI = body mass index.

**Table 3:** Neonatal Outcomes by Study Group.

Neonatal Outcome	No PE, Mild Anemia	No PE, Mod–Severe Anemia	PE, No Anemia	PE, Mild Anemia	PE, Mod–Severe Anemia
N	234	194	91	109	95
Birthweight (g)	3097 ± 405	2910 ± 437	2987 ± 416	2855 ± 423	2618 ± 461 *
Length (cm)	49.9 ± 2.0	48.9 ± 2.4	49.3 ± 2.1	48.8 ± 2.2	47.5 ± 2.6 *
Head Circumference (cm)	33.9 ± 1.3	33.1 ± 1.5	33.6 ± 1.3	33.2 ± 1.5	32.4 ± 1.6 *
Gestational Age at Birth (wks)	38.5 ± 1.4	37.8 ± 1.6	38.0 ± 1.6	37.4 ± 1.7	36.6 ± 2.0 *
Preterm Birth (%)	12.0%	21.6%	18.7%	29.4%	41.1% *
NICU Admission (%)	10.7%	16.5%	17.6%	28.4%	40.0% *
Apgar Score <7 (%)	2.6%	5.2%	3.2%	9.2%	12.6% *

Values are presented as mean ± standard deviation or percentage of group total. PE = preeclampsia; NICU = neonatal intensive care unit. * indicates statistically significant differences in pairwise comparisons: PE, Mod–Severe Anemia vs. PE, No Anemia: significant differences in birthweight, gestational age, head circumference, and all categorical outcomes (*p* < 0.001). PE, Mild Anemia vs. No PE, Mild Anemia: significant differences in birthweight, gestational age, and NICU admission (*p* < 0.001). PE, Mod–Severe Anemia vs. No PE, Mod–Severe Anemia: significant differences in birthweight and gestational age (*p* < 0.001). Apgar score <7 at 5 min was used as a clinically meaningful threshold for neonatal compromise. Mean and standard deviation were not reported, as Apgar score is an ordinal variable. Values are presented as mean ± standard deviation or percentage. Group PE, Mod–Severe Anemia shows the most adverse profile across all variables, consistent with compounded risk due to both preeclampsia and moderate-to-severe anemia.

## Data Availability

The raw data supporting the conclusions and results of this article will be made available on request.

## Abbreviations

The following abbreviations are used in this manuscript:

ACOG	American College of Obstetricians and Gynecologists
AUC	Area Under the Curve
BMI	Body Mass Index
CI	Confidence Interval
CRP	C-Reactive Protein
Hb	Hemoglobin
LMIC	Low- and Middle-Income Countries
NICU	Neonatal Intensive Care Unit
OR	Odds Ratio
PE	Preeclampsia
ROC	Receiver Operating Characteristic
SD	Standard Deviation
SMD	Standardized Mean Difference
SPSS	Statistical Package for the Social Sciences
STROBE	Strengthening the Reporting of Observational Studies in Epidemiology
WHO	World Health Organization
